# Preparation and characterization of a soy protein based bio-adhesive crosslinked by waterborne epoxy resin and polyacrylamide

**DOI:** 10.1039/c9ra05931h

**Published:** 2019-10-30

**Authors:** Zongtao Wang, Yuan Chen, Shiqing Chen, Fuxiang Chu, Ran Zhang, Yong Wang, Dongbin Fan

**Affiliations:** Research Institute of Wood Industry, Chinese Academy of Forestry Beijing City 100091 China fandongbin8@163.com +86-10-62881937 +86-18500236090; Institute of Forest Products and Industry, Hunan Academy of Forestry Changsha Hunan Province China

## Abstract

A simple and useful approach by using non-toxic and water-soluble raw material to improve the bonding properties of soy protein adhesive has attracted much attention recently. The objective of this research was to provide a simple and environmentally friendly approach for preparing a high adhesion performance soy protein adhesive in aqueous solution by using waterborne-epoxy resin, soy protein and water-soluble polyacrylamide (PAM). The chemical structure and curing characteristics, as well as the initial viscosity and adhesion performance of the resulted soy protein adhesive were characterized by ^1^H nuclear magnetic resonance (^1^H-NMR), differential scanning calorimetry (DSC), a rotary viscosity meter and a plywood panel test. The ^1^H-NMR analysis results confirmed that epoxy resin was successfully crosslinked with the –NH_2_ groups of the soy protein molecule to form a water-resistance network structure, and the resulting adhesive contains active epoxy groups. It was found that the addition of PAM can decrease the apparent viscosity, lower curing temperature, and enhancing the wet shear strength of soy protein adhesives effectively, which were capable of facilitating their application as wood adhesives. The resulting soy protein adhesive containing 4% epoxy resin and 0.05% PAM dosage had a reasonable viscosity and lower cure temperature, and showed good water resistance and wet shear strength, which met the requirement for interior use plywood of the Chinese Industrial Standard. These results suggested that waterborne-epoxy resin can be used to prepare high-performance environmentally friendly soy protein adhesives, which might provide a feasible methodology to prepare bio-adhesive adhesives for plywood industrial applications.

## Abstract 2

Waterborne epoxy resin mixed with polyacrylamide crosslinked modified soybean protein adhesive.

## Graphical abstract

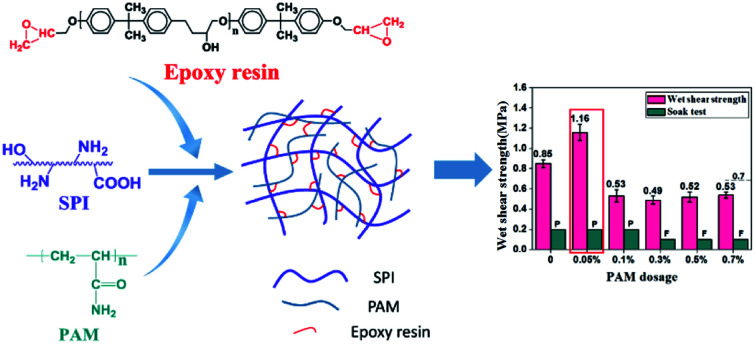

## 1. Introduction

Industrial interest in bio-based polymeric materials from renewable agricultural products has received increased attention over the past decade. Soy proteins are among the most investigated natural compounds for various industrial applications such as wood adhesives, food packaging, bio-based composites and coatings, *etc.* due to their plentiful supply, excellent properties and high functionality.^1^ In recent years, soy proteins have attracted great attention to prepare bio-adhesives for bonding wood composite materials.^2^ However, the low bonding strength and poor water resistance of soy protein bio-adhesives limited their more extensive application in high-performance wood adhesives. Soy protein, the main byproduct obtained from the production process of soybean oil, contains several functional groups, such as primary amine, hydroxyl and carboxylic groups.^3^ These reactive function groups make the soy protein molecule available to be modified by chemical approaches for preparing a competitive formaldehyde-free bio-adhesive. Thus, many efforts have focused on improvement in the adhesive properties by modifying the soy protein, mainly including chemical denaturation, crosslink modification, enzyme treatment, and protein molecular modification.^2^

The denaturation treatment of soy protein usually used urea, alkali, sodium dodecyl sulfate, and guanidine hydrochloride to unfold the complex molecule structure of protein and expose the internal hydrophobic groups to enhance the soy protein adhesives water resistance.^4–7^ However, the resulting molecule structure network of adhesive is relatively easy to hydrolyze in wet conditions, due to its contained many hydrophilic groups. For soy protein molecular modification, high activity groups were normally grafted onto soy protein molecules through esterification, acylation reaction, *etc.* to form a cross-linked network structure.^8,9^ This method can markedly improve the water resistance of soy protein adhesive, however, the main drawback is the complex preparation procedures, which makes it impractical for application in the plywood production.

Crosslinking modification is regarded as an effective and facile approach for improving the water resistance of soy protein adhesives. This modification mean use of different chemical species to increase the spatial density of potential crosslinking site. Active polymer and resin appear to be efficient and feasible cross-linkers, since their containing reactive groups can react with the hydroxyl, amino and carboxylic acid groups of soy protein molecular to form a covalent-linkages network within the protein matrix to improve the bonding properties and water resistance of soy protein adhesives. Several researchers have used polymeric methane diphenyl diisocyanate, polyamidoamine-epichlorohydrin (PAE) resin, polyethylene glycol adipate, synthetic latex and epoxide resins as cross-linkers to modify soy protein adhesives, and they have already been proven effective.^10–15^ In these cross-linkers, epoxy monomer and resulting resins have received special attention, because epoxy groups can freely react with abundant nucleophilic groups such as –NH_2_, –OH and –COOH on the soy protein molecular. Furthermore, most epoxy resins were produced from the epichlorohydrin, which can be derived from sustainable resource of bio-based glycerol.^16^ Huang J. *et al.* synthesized three types of polyepoxides including triglycidylamine (TGA), glycerol polyglycidyl ether (GPE) and trimethylolpropane triglycidyl ether (TTE) as crosslinker for soy-based adhesives.^17^ The plywood panels bonded by soy-based adhesives with TGA and GPE did meet the interior use requirement.

Commercial epoxy resin was also an effective cross-linker for enhancing the water resistant and bonding properties of soy protein-based adhesives. Luo *et al.* reported that a soy-based adhesive was prepared by using soybean flour, polyacrylamide, and epoxy resin as cross-linker, which met wet shear strength requirements of interior use panel.^18^ Enzyme-treated soy-based adhesive was blended with commercial epoxy resin before the application, and a water resistance crosslinked structure was formed during its curing process, resulting in the improvement of plywood bonding strength.^19^ In addition, the addition of epoxy resins to soy-based adhesives was found to improve its gluability and flowability. These efforts mainly focus on physical mixing of soy-based adhesive directly with cross-linker before its application, whose water-resistant structure was formed during its hot-press curing process. However, taking account of longer curing time of epoxy resin,^20^ the limited hot-pressing time was not enough for the sufficient crosslinking reaction between epoxy groups and functional groups of soy protein. Furthermore, epoxy resin is oil soluble, which made it difficult to adequately dissolve in water-based soy protein adhesives, thus resulting in the reduction of reinforcement efficiencies of epoxy resin and requiring a large dosage. Waterborne epoxy are dispersed in aqueous solution as colloidal particles, and amine is usually utilized as curing agent. More importantly, many waterborne epoxies are formulated without volatile solvents and use merely water as a diluent, which leading to ultra-low, or even zero, VOC paints and varnishes.^21^ Therefore, using waterborne-epoxy resin as crosslinking agent added during the preparation process of soy protein adhesive through effective crosslinking provide a simple and useful approach to form uniform and well-distributed bio-adhesive. To our knowledge, this approach to soy protein adhesives modification has not, to date, been reported.

In the present work, a series of soy protein bio-adhesive was developed using waterborne-epoxy resin as cross-linker and soy protein. Because the reactive functional groups on soybean protein molecule are limited, we introduced polyacrylamide (PAM) containing rich amino group into the adhesive matrix to increase crosslinking density through the multiple reaction of epoxy resin, soybean protein, and PAM. Effects of epoxy resin and PAM on the developed adhesive properties were investigated, and by measuring initial viscosity and wet shear strength of plywood verify the performance of soy protein adhesives were enhanced. Crosslinking reaction between waterborne-epoxy resin and soy protein was also confirmed by ^1^H nuclear magnetic resonance (NMR). Differential scanning calorimetry (DSC) was also employed to evaluate curing properties of the adhesives.

## 2. Materials and methods

### 2.1 Materials

Soybean protein isolate (SPI, ≥95% protein) was purchased from Baiwei Biological Technology Co., Ltd, Hebei, China. Defatted soy flour (SF) with about 52.2% of crude protein ground from defatted soy meal was purchased from Shandong San Wei Oil Group Co., Ltd. The remaining components in SPI are mainly moisture (≤4%), ash (≤3%), and fiber (≤1%). The SPI was ground to 200 mesh or more firstly. Sodium hydroxide (NaOH) and other chemical reagents were provided by Xilong Scientific Co., Ltd. Non-ionic polyacrylamide (PAM) with 1.2 × 10^7^ molecular weights was obtained from Ruida Purification Materials Co., Ltd., Henan, China. A commercial waterborne-epoxy resin of bisphenol-A (F0704, without curing agent, the storage life is 6 months, and should be stored in a dry and clean house) was purchased from Shenzhen Jitian CHEMICAL Co., Ltd. from Guangdong Province, which had a weight per epoxide (WPE) of 463–468, 50% of solid content and viscosity of 820–950 mPa s. Poplar veneer (400 mm × 400 mm × 1.7 mm, 10% moisture content) was supplied by Linyi, Shandong Province, China.

### 2.2 Preparation of soy protein adhesive

Soy protein adhesive crosslinked by epoxy resin (EPOXY-SPI) and PAM modified EPOXY-SPI (EPOXY-SPI-PAM) were prepared according to the following procedure:

First, for the pure SPI adhesive, 20 g of SPI and 170 g of distilled water were added to into a three-necked flask and stirred rapidly for 20 min at 40 °C water bath. And then the pH value of SPI slurry was adjusted to about 9.0 with 20% NaOH solution (w/w).

For the EPOXY-SPI adhesive, various mass fractions of epoxy resin (1%, 2.5%, 4%, 5.5% or 7% of the pure SPI adhesive mass, based on the solid content of epoxy resin) were added into the pure SPI adhesive and continued stirring 30 min at 45 °C. Final adhesive was then obtained after cooling reaction product temperature to 25–30 °C.

For the EPOXY-SPI-PAM adhesive, the same procedure and the same amounts of ingredients as EPOXY-SPI adhesive were used except that different concentrations of PAM aqueous solution were used to substitute for distilled water, and the addition amount of epoxy resin was set to 4%. The used amounts of PAM (0.05, 0.1, 0.3, 0.5 and 0.7 wt%) were determined by the relative weight percentage of the resulted adhesive.

### 2.3 Dynamic viscosity measurement

The viscosity of the fresh adhesives was measured with a rotary viscosity meter (Brookfield DV-II instrument, USA). The experiments were conducted using NO. 3 rotor at 25 °C with a spinning rate of 12 rpm. There were triplicate replications for each measurement and the average value of viscosity was shown Table 1.

**Table 1:** Initial viscosity of the EPOXY-SPI and EPOXY-SPI-PAM adhesives

Epoxy resin dosage^a^	Viscosity (mPa s)	PAM dosage^b^	Viscosity (mPa s)
0	19 753	0	9624
1%	16 159	0.05%	2148
2.5%	11 325	0.1%	2568
4%	9624	0.3%	2843
5.5%	8349	0.5%	3354
7%	6229	0.7%	3978

a The amount of epoxy resin dosage (calculated by solid content) added is based on the pure SPI adhesive mass.

b Mass ratio of PAM aqueous solution.

### 2.4 ^1^H-NMR measurement

The adhesive samples (about 20 mg) were dissolved in 0.5 mL DMSO-d_6_ for ^1^H-NMR analysis. All spectra of sample were acquired with a Bruker DRX-400 spectrometer using a 3.4 μs pulse width with a 2.0 s water pre-saturation delay. The chemical shifts of each spectrum were accurate to 0.01 ppm.

### 2.5 DSC analysis

The DSC analysis were conducted using a DSC 8000 (PerkinElmer, USA) instrument with PYRIS thermal analysis software. Around 5 mg of adhesive sample were sealed in alumina pans and scanning temperature ranged from 30 to 250 °C at a heating rate of 10 °C min^−1^ under nitrogen gas (20 mL min^−1^).

### 2.6 Preparation of plywood

Three-ply poplar laboratory plywood was prepared using the prepared soy protein adhesives in this study. A certain quality of adhesives were brushed on both faces of the veneer by an adhesive roller with glue spread of 18 mg cm^−2^. The adhesive-coated veneer was stacked between two uncoated veneers with the grain directions of two adjacent veneers perpendicular to each other. The stacked veneers were stored for 10 min at ambient conditions, and then pressed at room temperature at 0.85 MPa for 5.5 min, followed by hot-pressing under 0.95 MPa at 120 °C for 6.5 min. The resulted plywood samples were stored under ambient conditions for at least 48 h before the wet shear strength and water resistance were tested.

### 2.7 Water resistance and wet shear strength measurement

The water resistance of interior use plywood samples were tested according to China National Standards for Type II Plywood (GB/T 9846-2015). More specifically, 6 specimens (75 mm × 75 mm) were cut from two plywood panels, and then soaked in 63 ± 3 °C tap water for 3 h; followed by drying at 63 ± 3 °C for 3 h. After this procedure, the specimens were inspected for delamination. As described in standard, a specimen fails if there is any continuous opening between two layers that is longer than 25 mm. A plywood panel passes water-resistance test for interior applications if 90% of the specimens do not delaminate after the above water-soaking procedure.

The test for wet shear strength was also made in accordance with GB/T 9846-2015. The plywood specimens (25 mm × 100 mm) were soaked in tap water at 63 ± 3 °C for 3 h, and then cooled down at room temperature before testing. At least twelve parallel samples from three different panels were measured and their mean value was reported as wet shear strength in Fig. 4.

## 3. Results and discussion

### 3.1 Viscosity analysis

Viscosity is very important property for the fabrication of plywood, which mainly governs the adhesive behavior including penetrability, flowability, *etc.* during manufacture.^22^ Generally, the high viscosity of soy protein adhesives may cause excessive amounts of adhesives left at the surface with little penetration into the pores; if low-viscosity adhesives are used, it will easily penetrate deep into wood through the veneer surface, so that little adhesive is left on the surface to bond the wood blocks. Either scenario results in low bond strength. Thus, the recommended viscosity of soy protein adhesive is around 6000 to 20 000 mPa s for sprayline coaters.^23^ According to the findings shown in Table 1, the apparent viscosity of pure SPI and EPOXY-SPI adhesives showed relatively low viscosity compared to other soy protein adhesives,^19^ which indicates good flowability and penetrability into the veneer, The introduction of epoxy resin led to a marked decrease in the viscosity of SPI adhesive, which agrees with a previous study.^18^ With the additive amount of epoxy resin, the viscosity of EPOXY-SPI adhesive gradually decreased by 18.1% from 19 753 to 16 159 mPa s, and further decreased by 68.5% to 6229 mPa s when 7 wt% of epoxy resin was loaded, which met the operating viscosity requirements of soy protein adhesive. One reasonable interpretation for this result was that waterborne-epoxy resin acted as a dispersant to reduce the viscosity of SPI adhesive. Due to the limited amino groups of soybean protein molecule,^24^ PAM was incorporated into the adhesive matrix to increase the amino group content for increasing the crosslinking degree of adhesive networks through the reaction of epoxy group and amino group. As can be seen from Table 1, all EPOXY-SPI adhesive with a certain amount of PAM addition showed lower viscosity than the EPOXY-SPI, attributed to the water-solubility and self-lubrication property that can decrease frictional resistance of the resulted adhesive molecules. Therefore, EPOXY as a crosslinker together with PAM seem to result in a low viscosity that was beneficial for handling and coating of the adhesive.

### 3.2 ^1^H NMR analysis

**Fig. 1:**
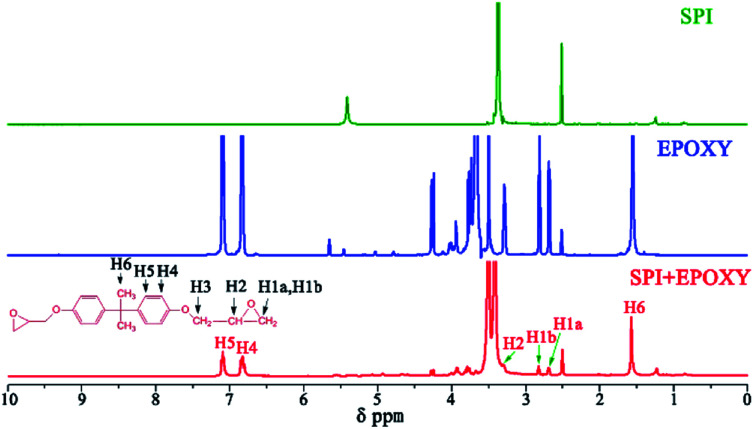
The ^1^H-NMR spectra of different adhesives.

**Fig. 2:**
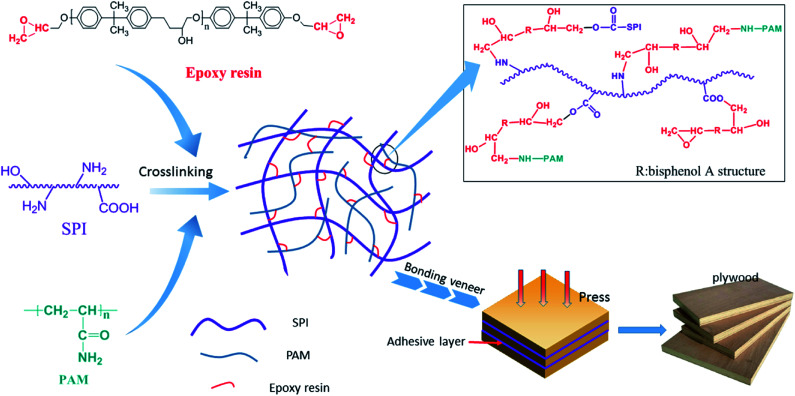
The reaction mechanism among of SPI, epoxy resin and PAM.

The appearance of new chemical shifts in ^1^H-NMR spectra is powerful evidence regarding whether or not crosslinking reaction between epoxy resin and SPI takes place during the preparation process of EPOXY-SPI adhesive. Therefore, ^1^H-NMR analysis was performed, and the spectra of the SPI, EPOXY resin and EPOXY-SPI adhesive are shown in Fig. 1, which is in consistent with reported analytical data for similar structures.^25–27^ As shown in Fig. 1, the spectra of SPI and EPOXY-SPI are obviously different. Compared to the former, some new peaks appeared in the EPOXY-SPI spectrum, which also were presented in EPOXY resins spectrum at the same chemical shift. The peaks in the region of *δ* 3.50–4.27 ppm can be principally assigned to the hydrogens of methylene and methyne on repeating molecular units of the bisphenol A structure. The peaks at *δ* 6.8 ppm and 7.1 ppm were attributed the hydrogens on the *ortho*- and *meta*-carbon of the benzene ring. An obvious peak at around 1.6 ppm was assigned to hydrogen on the methyl group. The signals at *δ* 2.61, 2.79 and 3.30 ppm were attributed to the protons of –CH_2_ and –CH in the epoxy group. Meanwhile, the peaks intensity of the epoxy group become much weaker compared with the epoxy resin, which might be a result of the consumption on some amount of epoxy group *via* the crosslinking reaction of epoxy groups with –NH_2_ and –COOH groups on the SPI molecules.^28^ These results above indicated that the epoxy groups of epoxy resin were able to react with the active groups of SPI molecules (*e.g.*, –NH_2_) during adhesive synthetic process by a ring opening reactions of epoxy groups, and that the resulting adhesive contained a certain amount of epoxy groups, which can further react with hydroxyl group of wood to form better bonding property during its curing process. The incorporation of epoxy resin into macromolecular structure of SPI tended to increase the crosslinking density of EPOXY-SPI adhesive matrix by chemical reaction and enhance its water resistance that was proved by the results in Fig. 4. The likely reaction mechanism was presented in Fig. 2.

### 3.3 Cure characteristics

**Fig. 3:**
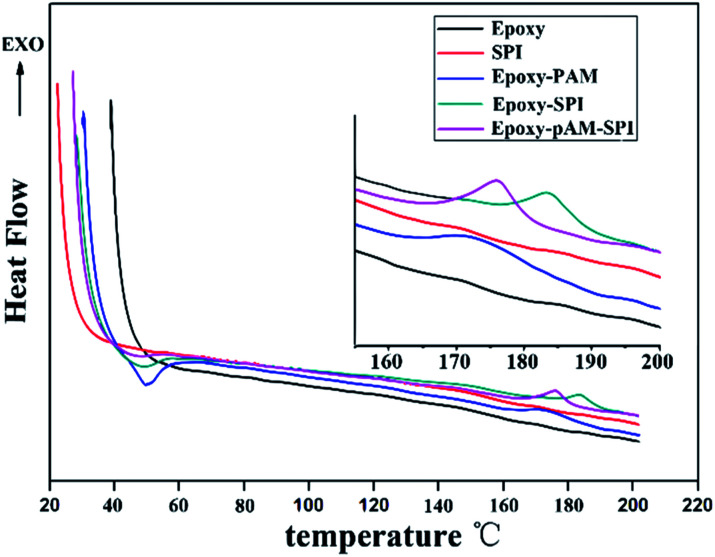
DSC curves of different soy protein adhesives.

The characteristic temperature range of the DSC curves gives valuable information on the cure reaction of resin adhesives. The curing properties of soy protein-based adhesives with or without crosslinker has been studied through DSC analyses, as shown in Fig. 3, and their results are presented in Table 2. It could be seen that there was no obvious exothermic peak for SPI and epoxy resin, indicating that SPI or EPOXY would not be crosslinked by itself. As it results from the ^1^H-NMR analysis spectra, EPOXY-SPI adhesive had a certain amount of epoxy groups, which was able to react further with amino groups of soy protein molecule or hydroxyl group of wood during curing. For the samples of EPOXY-SPI and EPOXY-PAM, one distinct exothermic peak appeared at temperatures of approximately 160–180 °C, thus attributed to curing reaction of epoxy groups with amino groups deriving from either the soy protein molecule or PAM polymer. It is also noted that the DSC exothermic thermograms of the EPOXY-SPI-PAM adhesive also displayed one peaks at 160–180 °C. Since SPI would not cure by itself without epoxy resin, this exothermic peak was caused by the cross-linking reaction of both soybean protein and PAM with epoxy resin. In this study, the addition of PAM to EPOXY-SPI caused an increase in the proportion of amide groups in the EPOXY-SPI-PAM adhesive system. As observed from Fig. 3, the EPOXY-SPI-PAM adhesive shifted down to lower curing temperatures compared to the pure SPI adhesive, a little higher than that of EPOXY-SPI sample. This results indicated that PAM addition accelerated the adhesive cure at a low temperature. It is possible that the formation of EPOXY-SPI-PAM adhesive networks increased the cross-linking density of the adhesive matrix, resulting that adhesive just need fewer steps to finish crosslink-curing reaction. Therefore, the curing temperature correspondingly decreased. A lower curing temperature of soy protein adhesive plays a primary role in producing plywood panels.^29^ The addition of PAM affected the Δ*H* value of EPOXY-SPI adhesives significantly. Table 2 shows the Δ*H* values based on the mass of liquid adhesive. The EPOXY-SPI-PAM adhesive has much higher Δ*H* value than the EPOXY-SPI adhesive, which suggested that the addition of PAM made EPOXY-SPI adhesive reacted more strongly because of the higher released Δ*H* during curing.^30^

**Table 2:** DSC curing data of the different samples^a^

Sample	*T*_i_ (°C)	*T*_p_ (°C)	Δ*H* (J g^−1^)
Epoxy	—	—	—
SPI	—	—	—
Epoxy-PAM	161.37	171	12.48
Epoxy-SPI	175.07	185	10.74
Epoxy-SPI-PAM	164.18	173	16.21

a *T*_i_: Onset curing temperature; *T*_p_: peak curing temperature; Δ*H*: enthalpy value.

### 3.4 Wet shear strength and water resistance

#### 3.4.1 Effects of epoxy resin dosage on the wet shear strength and water resistance

**Fig. 4:**
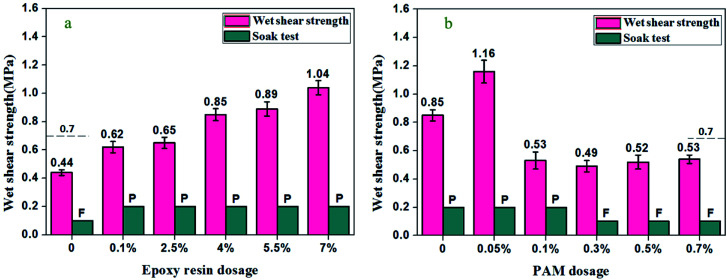
Wet shear strength and water resistance of different adhesives. (a) EPOXY-SPI adhesive with different epoxy resin dosages; (b) EPOXY-SPI-PAM adhesive with different PAM dosages. P: pass the soak test, F: fail the soak test.

The wet shear strength and water resistance of EPOXY-SPI adhesives with different amounts of epoxy resin are presented in Fig. 4. The wet shear strength of plywood bonded with pure SPI adhesive without epoxy resin is 0.44 MPa, which did not reach the threshold value of 0.7 MPa required for interior use plywood. As can be clearly seen, the shear strength of all adhesives increased with increase of epoxy resin addition amount, indicating that the bonding performances of the resulted adhesives were improved by epoxy resin. Two reasonable interpretation for this were probably that: (1) epoxy groups of epoxy resin were capable of reacting with the amino and hydroxyl groups to form three-dimensional crosslinked network structure of the resulted adhesives;^19,28^ (2) the hydrophilic groups (–COOH, –NH_2_) of soy proteins were consumed by epoxy group to increase relative hydrophobic property. When the addition amounts of epoxy resin were increased to/or more than 4 wt%, the shear strengths were higher than 0.70 MPa, which met the GB/T 9846-2015 standard requirements for interior use plywood. Although the EPOXY-SPI adhesive with 2.5 wt% epoxy resin had not satisfactory wet shear strength (0.65 MPa), it still pass the water resistance test. By increasing the epoxy resin additive amount to 7 wt%, the shear strength reached the maximum value of 1.04 MPa, which about 136% increase compared with pure SPI adhesive. These results show better reaction between epoxy resin and soy protein to form a water resistance structure, which was also supported by ^1^H-NMR analysis. On the other hand, the viscosity of EPOXY-SPI adhesives obviously reduced (the results were showed in Table 1) after adding epoxy resin, which made the adhesives favorable to penetrate into the veneer surface. Thus, forming better mechanical interlocking and efficient interfacial contact, which further increased the shear strength of the plywood specimens. The water resistance of plywood bonded with different adhesives were also shown in Fig. 4(a). SPI is water soluble polymer which contains a large number of hydrophilic groups, leading to poor water resistance of the pure SPI adhesive bonded plywood.^1^ The specimens bonded by the EPOXY-SPI adhesive with 1 wt% epoxy resin was delaminated, which did not pass water resistance test. A possible reason was that the addition amount of epoxy resin was not sufficient to form water-resistant network. However, after increasing the addition amounts of epoxy resin to 2.5–7 wt%, all the specimens had good water resistance, meeting GB/T 9846-2015 regarding water resistance requirement. Therefore, based on the shear strength value and cost of the resulted soy protein adhesives, the optimum dosages of epoxy resin was 4 wt%.

#### 3.4.2 Effects of PAM dosage on the wet shear strength and water resistance

In this work, PAM was introduced into EPOXY-SPI adhesive for further increasing the crosslinking degree of adhesive networks. With the aim to optimize the reasonable dosage of PAM, the EPOXY-SPI adhesive with 4 wt% epoxy resin was chosen for further investigation. Effect of the dosage of PAM on shear strength and water resistance of EPOXY-SPI adhesive, the results are presented in Fig. 4(b). As can be seen, the wet shear strength obviously increased after introducing PAM to EPOXY-SPI adhesive and reached the maximum value of 1.16 MPa at 0.05 wt% PAM concentration for the EPOXY-SPI-PAM adhesive, and then decreased to the value below 0.70 MPa at higher PAM concentration, which indicated that the resulted adhesives with more than 0.1 wt% PAM dosage did not be used for industrial application of interior use plywood. This wet shear strength value of 1.16 MPa was comparable to that of the formaldehyde-based resin adhesives,^31^ which was also slightly higher than that of soybean based adhesive modified by 5 wt% epoxy resin in previous research,^18^ however, our approach to prepare soy protein based adhesive used relatively lower dosage of epoxy resin. These comparative analysis indicated that waterborne-epoxy resin appeared to be a high-efficient crosslinker for enhancing water-resistant structure of soy protein adhesives because of its good water-solubility. In general, an increase in crosslinking degree of cured soy protein bio-adhesives would be expected to improve its water resistance and bonding strength. PAM is a water-soluble polymer including many amino groups, therefore, degree of crosslinking can be further increased by the multiple reaction of epoxy resin, PAM, and soybean protein.^32^ This reason was mainly responsible for significant improvement applications. Furthermore, since all raw materials used in the waterborne-epoxy resin crosslinked soy protein adhesive were water-solubility, it would be non-toxic and environmentally safe.

#### 3.4.3 Effects of epoxy resin dosage on the wet shear strength and water resistance of soy flour based adhesive

**Fig. 5:**
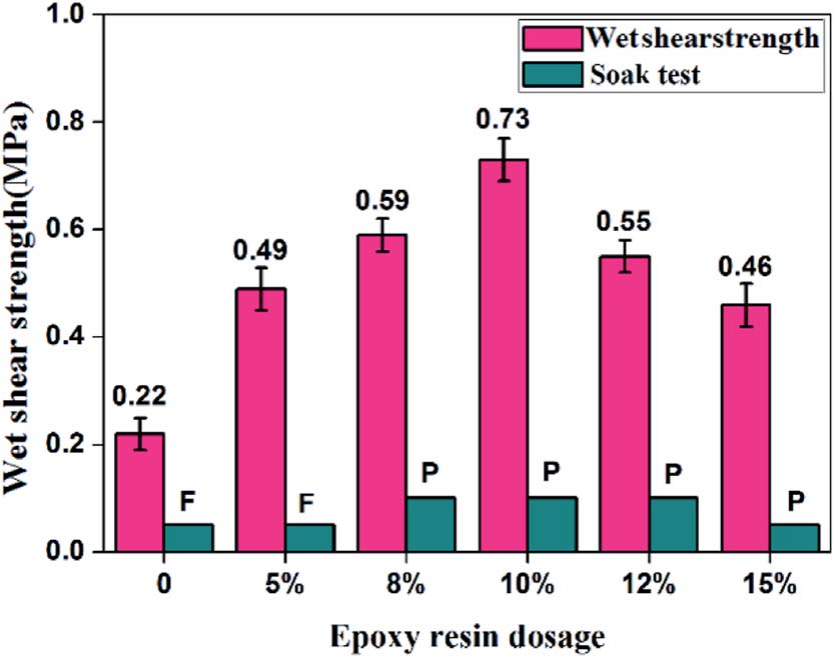
The wet strength and water resistance of EPOXY-SF-PAM adhesive. P: pass the soak test, F: fail the soak test.

We noted that SPI is more expensive than soy flour (SF), therefore, SF is used to replace SPI for preparing soy protein adhesive (EPOXY-SF-PAM) and the preparation method is similar to that of SPI adhesive except for the raw materials. In this work, the effects of different epoxy resin dosage on bonding properties of EPOXY-SF-PAM adhesives were investigated. The wet shear strength of EPOXY-SF-PAM adhesives are shown in Fig. 5. The wet shear strength of plywood bonded with pure SF adhesive without epoxy resin was 0.22 MPa, which was only half value of pure SPI adhesive. This reason could be ascribed to high content of water-solvent polysaccharide of SF. After adding the waterborne epoxy resin, the wet shear strength increased significantly and reached the maximum value of 0.73 MPa when epoxy resin dosage was 10 wt%, which met the requirement for interior use plywood. However, with an increase in epoxy resin dosage, the value of wet shear strength decreased, which might result from the addition of epoxy resin is excessive. Excessive epoxy resulted in some residues in the cured adhesive, thus making a drastic decrease in the shear strength, due to the existence of free epoxy groups are sensitive to water. In conclusion, waterborne-epoxy resin combined with polyacrylamide cross-linked modified soybean protein adhesive has a significant effect on enhancing bonding property and practical production value.

## 4. Conclusions

In this work, an environmentally friendly soy protein bio-adhesive was developed using waterborne-epoxy resin, soy protein, and water-soluble PAM. Epoxy resin was crosslinked with the active groups of soy protein as well as PAM to form a water-resistance network structure through their multiple reactions. ^1^H-NMR analysis results also indicated that the resulting adhesive contain a certain amount of epoxy groups, which can further react with hydroxyl group of wood to form better bonding property during its curing. It is also seen that the addition of PAM to EPOXY-SPI was capable of decreasing the cure temperature and increasing the wet shear strength of soy protein adhesives. The crosslinked adhesive with the dosage of 4 wt% epoxy resin and 0.05 wt% PAM showed better wet shear strength and water resistance property. When the epoxy resin addition increase from 5 wt% to 10 wt%, the property of EPOXY-SF-PAM adhesive is also meet requirement of interior use plywood. In view of the stringent formaldehyde emission limits imposed on interior-used plywood, the waterborne-epoxy resin crosslinked soy protein adhesive is a better substitute as wood adhesive for formaldehyde-based resins.

## Conflicts of interest

There are no conflicts to declare.

## Supplementary Material
